# Development of a remote learning educational model for international Emergency Medicine trainees in the era of COVID-19

**DOI:** 10.1186/s12245-021-00405-1

**Published:** 2022-01-06

**Authors:** Joseph D. Ciano, John Acerra, Aimee Tang

**Affiliations:** Department of Emergency Medicine, NSLIJ Health System: Northwell Health, Queens, New York, USA

**Keywords:** Global Emergency Medicine, Remote education, LMICs, Emergency Medicine specialty development

## Abstract

**Background:**

The COVID-19 pandemic has pressured post-graduate medical education programs to shift from traditional in-person teaching to remote teaching and learning. Remote learning in medical education has been described in the literature mostly in the context of local in-country teaching. International remote medical education poses unique challenges for educators, especially in low-middle income countries (LMICs) who need continued Emergency Medicine (EM) specialty development. Our objective is to describe the development and implementation of our remote educational curriculum for EM trainees in West Bengal, India, and to assess trainee satisfaction with our remote learning curriculum.

**Methods:**

Our curriculum was developed by adapting remote learning techniques used in Western post-graduate medical education, conducting literature searches on remote learning modalities, and through collaboration with local faculty in India. We assessed resident satisfaction in our curriculum with feedback surveys and group discussions.

**Results:**

The remote educational curriculum had overall high trainee satisfaction ratings for weekly livestream video lectures and throughout our monthly educational modules (median ratings 9-10 out of a 10-point Likert scale). Qualitative feedback regarding specific lecture topics and educational modules were also received.

**Conclusions:**

International remote education in LMICs poses a unique set of challenges to medical educators. Residents in our study reported high satisfaction with the curriculum, but there is a lack of clarity regarding how a remote curriculum may impact academic and clinical performance. Future studies are needed to further evaluate the efficacy and academic and clinical implications of remote medical education in LMICs.

## Background

The first documented cases of COVID-19 were recorded in December 2019 in Wuhan, China [1]. Since the identification of the initial cases of the virus, COVID-19 has spread throughout the world and impacted all facets of life, including medical education. The COVID-19 pandemic has required medical educators across the globe to adapt their teaching approach from in-person teaching to remote teaching. Educational didactics for medical and surgical residencies have shifted to remote learning conferences in order to maintain social distancing and avoid large gatherings [2–5]. Remote learning is conducive to the use of technology-enhanced modalities, which prioritize active learning, encourage learner independence, and help foster educational accountability [1, 6]. While in-person patient encounters serve as clinical education for resident physicians, there is also a role for formal didactics to enhance clinical development [7]. It also is unrealistic for post-graduate medical education programs to pause education for residents, as this would be disruptive to the overall structure of their residency education [2].

Emergency Medicine was recognized as a medical specialty in India in 2009 [8]. Prior to government recognition, a variety of short training courses, education conferences, and certification courses were created to help physicians in India gain knowledge in Emergency Medicine [8, 9]. Masters in Emergency Medicine (MEM) Certification courses, which often run in partnership with US institutions with experience in emergency medicine training, are one example of the effort to help bridge the gap between the country’s lack of Emergency Medicine trained doctors and the country’s need for doctors with this specialized training [9, 10].

Since 2009, our home institution has run a 3-year MEM Certificate program in partnership with a corporate hospital in Durgapur, West Bengal, India. The pre-COVID-19 model for this program included monthly, in-person visits from our US-based faculty for both didactic training and bedside teaching in the emergency department (ED). The COVID-19 pandemic has caused travel lockdowns which prevented any physicians from our home institution in New York, USA, from traveling to India to continue this important educational work. COVID-19 has also affected transportation in India which posed challenges for students in the course to get to work to receive education from local faculty. The objective of this manuscript is to describe our development and implementation of a remote educational curriculum for Emergency Medicine trainees in West Bengal, India, and to assess trainee satisfaction with the remote learning curriculum. We hope in sharing our model we can offer a framework which can be replicated in other countries that would benefit from a remote learning model.

## Methods

This project was submitted to our IRB, and it was deemed to be exempt from review as it did not meet the definition for human subject research. The development phase of our remote learning curriculum required course faculty to meet remotely to discuss ideas and common goals regarding the curriculum. Topics that were discussed during curricular development included types of educational content, methods to deliver educational content, challenges to implementing a remote curriculum in a LMIC, ways to incorporate gathering feedback from the students throughout the curriculum, and a tentative start date and schedule. Some LMIC-specific challenges that were discussed included potential issues with WiFi or data connectivity during lectures, finding a balance between trainee education and hospital needs, and maintaining flexibility in our program as resident workforce needs were anticipated to increase during COVID-19 surge capacity times. Our training site was able to provide WiFi for teaching purposes, so this was not a barrier in implementing our remote program. Residents also utilized data on their personal mobile phones to log into live video lectures and access asynchronous learning content. The West Bengal Emergency Department had baseline low physician staffing at the time of the study which placed high reliance on the resident workforce for patient care. This required coordination with local faculty in order to find ideal lecture times during which the department had lower patient volumes. We collaborated with our home institution’s core education faculty to brainstorm ideas to use in the curriculum and through literature searches on remote learning modalities. All decisions made during the curriculum development phase were agreed upon by shared consensus and in close collaboration with local teaching faculty in West Bengal, India.

All teaching materials were organized to fit into the residents’ 36-module curriculum which was already established in the MEM certification course (Table 1).

**Table 1:** Master in Emergency Medicine certification program educational modules (executed over 36 months)

Orientation	Obstetrics/Gynecology	Airway
Trauma I	Endocrine	Shock
Neurology	HEENT	Infectious Disease II
Orthopedics I	Hematology/Oncology	Trauma II/Orthopedics
Cardiology I	Toxicology I	Pediatrics II
Pediatrics I	EMS and Disaster	Soft Tissue/Wound Care
Pulmonary	Environmental	Cardiology Critical Care
Gastrointestinal	Procedures	Radiology
Psychiatry	Immunology/Rheumatology	Orthopedics II/Trauma
Infectious Disease I	Research	Toxicology II/Tropical
Genitourinary/Renal	Orientation/Review	Administrative/Public Health
Dermatology/Ophthalmology	Cardiology II	Review/Oral Boards

In this template, each 4-week module covers a topic relevant to Emergency Medicine. Each module lists monthly objectives, recommended reading content for asynchronous learning, and lists in-person lecture topics and times. We adapted this template by including a variety of recommended audio, video, and other online content along with weekly live remote video lectures on Zoom software. Each week of the module provided educational resources for students to use with faculty facilitation and resources for students to use independently. Educational resources that were used under US-faculty facilitation consisted of once weekly live Zoom class sessions and team-building games. Some of the instructors in the Zoom sessions were visiting faculty members with whom the residents were already familiar, as well as some new lecturers who volunteered from our home institution. A comparison between our pre-COVID-19 in-person curriculum and remote learning curriculum is summarized in Table 2.

**Table 2:** Comparison of MEM educational curriculum prior to COVID-19 and during COVID-19 pandemic

	Curriculum prior to COVID-19 pandemic	Curriculum during COVID-19 pandemic
**Location of teaching**	100% in-person (India)	Combined in-person (India) and remote
**Educational topic covered each month** (i.e., Cardiology, Pulmonology, etc.)	Based on 36-month modular schedule	Based on 36-month modular schedule
**Required monthly readings in Tintinelli’s Emergency Medicine text**	Yes. Readings based on topic covered monthly.	Yes. Readings based on topic covered monthly.
**Educational questions assigned monthly from purchased question bank**	Yes	Yes
**In-person lectures by local faculty** (40–60 min)	Yes (once weekly)	Yes (once weekly)
**Virtual lectures by local faculty**	No	No
**In-person lectures by international faculty** (40–60 min)	Yes (given over 1 week time period during monthly in-person visits)	None provided due to travel restrictions imposed by COVID-19
**Virtual lectures by international faculty** (40–60 min)	No	Yes. One live lecture given weekly via Zoom software
**Asynchronous FOAM-ed content provided** (videos, audio podcasts, and interactive “click-through” medical cases)	No	Yes. Provided as supplement to in-person lectures, remote lectures, and readings

Class sessions varied from 40 to 60 min in length and were constructed with a focus on high-yield clinical content. Lectures were often case-based and interactive, requiring input from students throughout the session. This lecture style was similar to the in-person teaching style used prior to the COVID-19 pandemic. Team-based Jeopardy-style games and the use of audience response systems were also integrated into our monthly Zoom lecture time. The weekly materials for independent student use were chosen from varying modalities to appeal to different learner types. Some examples of the types of educational materials we utilized include pre-recorded video lectures, videos on the use of point of care ultrasound, short readings, click-through medical and electrocardiogram cases, and educational audio podcasts. Integration of these modalities was novel in our remote curriculum, as only in-person teaching and required monthly readings were included in our original curriculum (pre-COVID-19). The majority of the non-lecture content was curated from Free Open Access Medical Education (FOAM-ed). Sources of FOAM-ed we used are listed in Table 3.

**Table 3:** Sources of Free Open Access Medical Education (FOAM-ed) used in our curriculum

▪ Dr. Smith’s ECG Blog ▪ Life in the Fast Lane (LITFL) ▪ 5 Minute Sono ▪ CORE-EM ▪ REBEL-EM ▪ EMCRIT ▪ Emergency Medicine Cases ▪ FOAM-cast ▪ Corependium ▪ Pediatric EM Morsels ▪ EMPEM ▪ Don’t Forget the Bubbles	

Residents were provided additional sources of education through assigned questions from a purchased question bank (ROSH review) and weekly in-person lectures by their local teaching faculty. Educational questions and in-person lectures by local faculty were not new components of our established MEM curriculum.

We distributed electronic feedback surveys to the residents following each live Zoom lecture and at the end of each 4-week educational module. Monthly feedback surveys were also distributed to residents prior to the implementation of our remote learning curriculum. These pre-COVID-19 surveys focused on evaluation of the visiting international faculty rather than the evaluation of specific lectures or the curriculum at large.

During the 5-month study period, surveys were distributed following each weekly class session and following each 4-week educational module. Surveys were strongly encouraged but not made mandatory to complete. Reminder emails were sent after surveys were initially distributed to promote survey completion. The survey was specifically developed by the authors to assess learner satisfaction with the remote learning curriculum. The survey was comprised of five 10-point Likert items where residents rated their satisfaction with the lecture and content, as well as a free text area where residents could leave qualitative feedback. This information was used to continually adapt details of the remote curriculum to meet the educational needs of the students.

## Results

After several weeks of planning the details of the new curriculum, we were able to develop and implement a new remote learning curriculum for Emergency Medicine resident physicians enrolled in our MEM certification program. In-person lectures from visiting faculty members were replaced by weekly live video lectures and a variety of educational activities for independent resident study. Other faculty members from our home institution were recruited and engaged in our program to assist in our remote educational efforts.

Results from our feedback surveys indicated overall high levels of satisfaction with the individual Zoom lectures given and the modules at large. These results represent average ratings on the 10-point Likert item reported by the 9 resident physicians to whom the surveys were distributed. Overall satisfaction ratings for the lectures were rated as 9.24 (mean), and overall lecture relevance to clinical work was rated as 9.28 (mean). One crucial aspect of our remote education model was independent learning with the video, audio, and other educational modalities provided. Residents were given this independent learning material in monthly modules, but they needed to access the material during their free time. Resident physicians rated the degree to which they used independent educational content as 9.40 (mean). The degree to which this independent educational content was found useful to learning was rated as 9.40 (mean).

Overall satisfaction with the modules was rated by trainees as 9.20 (mean). Team-based activities, like Jeopardy-style review games, were rated as 9.25 (mean). These activities were typically offered towards the end of modules as an opportunity to review the content of each module. Quantitative survey results are further detailed with their mean and median values in Table 4. Selected qualitative feedback provided by the residents can be found in Table 5.

**Table 4:** Quantitative results for feedback surveys using a 10-point Likert scale (1 = not at all satisfied, 10 = very satisfied)

**Overall satisfaction rating of lectures** (*n*=25)	
Mean = 9.24 Median = 10.0	
**Rating of overall lecture relevance to clinical work** (*n*=25)	
Mean = 9.28 Median = 9.0	
**Overall satisfaction rating of educational modules** (*n*=15)	
Mean = 9.20 Median = 9.0	
**Rating for degree to which educational materials provided in modules were useful to learning (i.e. audio podcasts, videos, click-through cases)** (*n*=8)	
Mean = 9.40 Median = 10.0	
**Overall satisfaction rating for team-based activities used in the modules (i.e. Jeopardy-style game)** (*n*=8)	
Mean = 9.25 Median = 9.5

**Table 5:** Qualitative feedback provided by residents

“Pls give us more ECG examples…” “It should be more case based study…” “Everything was excellent!!!” “You may involve all of the students by questioning them after giving a case scenario…then discuss…and question everyone…thank you” “Satisfied…. thanks” “The presentation should be complete with details Clinical feature how to diagnose and treatment with new guidelines”	

## Discussion

Our remote learning project was well received by our Emergency Medicine trainees with high reported overall satisfaction ratings. This is relevant as remote learning has been the standard approach to post-graduate medical education during the COVID-19 pandemic [2–5]. However, much of the literature that describes using this approach refers to local in-country graduate medical education with little consideration for the nuances and challenges to implementing remote learning in international medical education. The importance of continued medical education and specialty development becomes especially profound when dealing with low-middle income countries (LMIC), such as India, where Emergency Medicine specialty development is still in its early stages [8].

The presence of reliable and organized local faculty to help coordinate educational efforts is crucial. Without effective local faculty, or a resident doctor liaison, carrying out a remote curriculum becomes difficult. Remote teaching in a LMIC also poses its own obstacles. A major hurdle in some LMICs is a lack of resources. These resources can be people, infrastructure, institutions, or technologies. Fortunately, our teaching site has local faculty, an established infrastructure, and access to wireless internet, computers, a projector, and other resources that made the implementation of our remote learning curriculum easier. We acknowledge that not all medical education sites in LMICs possess these same technologies and resources. Reliance on more independent learning and assigned educational content may be necessary in these settings.

Remote learning curricula can be integrated with active learning educational modalities. We created our curriculum with student engagement in mind and promoted active learning principles throughout our curriculum. Our results of high resident satisfaction mirror those seen in previous studies assessing learner perception of the flipped classroom [11, 12]. However, more research needs to be conducted to determine if remote learning changes outcomes in academic performance.

Time zone difference is another factor that needs to be considered when designing an international remote learning curriculum. West Bengal is 9.5 hours ahead of the Eastern Daylight time zone where our faculty live, so live class session times were limited to early morning or late evening. Depending on the time zone differences between the two parties involved, this can be a significant barrier to live lectures. Pre-recorded lectures and the use of other FOAM-ed resources may be helpful in these situations, although this may have implications on resident learner satisfaction.

Our typical in-person teaching includes interactive simulated cases and supervised procedural practice (i.e., endotracheal intubation) with mannequins. One constraint of a virtual educational model is that in-person simulation activities are unable to be included as part of the model. Some simulation activities are more amenable to be carried out virtually, such as oral board review cases. Virtual procedural simulation can include video instruction followed by hands-on procedural practice, but this requires closer partnership with local faculty to gather materials and directly observe trainees for appropriate performance.

## Limitations

Our project has three main limitations. The first limitation pertains to uncertainty regarding the impact of the remote curriculum on academic and clinical performance. We have developed our remote curriculum out of necessity, but we do not know the implications that this new mode of teaching will have, if any, on resident academic or clinical performance. Our data shows high resident satisfaction, however, satisfaction does not mean this method of teaching is as effective as traditional in-person teaching. Further research investigating the effectiveness of remote medical education on academic and clinical performance is needed to better understand these relationships.

The second limitation of our project is size and limited power in the quantitative results. Our MEM certification course program currently has a total of 9 enrolled physicians throughout all resident classes. This small group size made the implementation of the curriculum more manageable, but it limits the generalizability of our results. Surveys being optional to complete may have created selection bias in the results, as residents more satisfied with the curriculum may have been more interested to complete the surveys and express positive feedback in order to ensure the curriculum would continue. Additionally, the survey we used is not validated or standardized for assessing resident satisfaction. The quantitative results we present here reflect five months of resident responses. Implementation of our remote curriculum is ongoing, but we felt it important to present the results we have accumulated to share a fuller picture of resident satisfaction with our curriculum.

The third limitation of our project is the lack of a control study group. The feedback surveys used during pre-COVID-19 teaching and remote teaching times had different focuses, so the questions were different and could not be directly compared. The absence of a control group makes it difficult to ascertain if the residents’ satisfaction ratings with the remote curriculum are worse, similar, or better than satisfaction with the original in-person learning curriculum. Prior to the pandemic, resident evaluations showed high satisfaction with the quality of faculty we were sending to teach in person. With the new curriculum, residents were satisfied with the quality of the remote learning instruction.

## Conclusion

We have successfully developed and implemented a remote learning curricular model for international Emergency Medicine trainees. Our study demonstrates that trainees have used this curriculum with high levels of overall satisfaction. Future studies are needed to further investigate the topic of remote medical education in LMICs and their effectiveness.

## Abbreviations

LMICs: Low-Middle income countries
EM: Emergency Medicine
MEM: Masters in Emergency Medicine
FOAM-ed: Free Open Access Medical Education
